# Impact of Season on Chemical Composition of Some Medicinal Plants in Saudi Arabia

**DOI:** 10.3390/life15030336

**Published:** 2025-02-21

**Authors:** Deema A. AlZunaydi, Abdulaziz B. Alharbi, Ahmed H. Alfarhan

**Affiliations:** 1 Botany and Microbiology Department, College of Science, King Saud University, P.O. Box 2460, Riyadh 11451, Saudi Arabia; 2 Department of Biology, College of Science, Qassim University, Buraydah 52454, Saudi Arabia; 3 Department of Environment and Natural Resources, College of Agriculture and Food, Qassim University, Buraydah 52454, Saudi Arabia; abb.alharbi@qu.edu.sa

**Keywords:** seasonal fluctuations, medicinal plants, nutrient content, primary metabolites, secondary metabolites, soil proprieties

## Abstract

Wadi Al-Rummah is one of the most important geographical phenomena in the Najd region of Saudi Arabia and is considered to be the largest and longest valley in the Arabian Peninsula, with most of its basin located in the Qassim region. This valley is the habitat of diverse flora, including medicinal herbs, plants, and trees. Three plant species, namely, *Capparis spinosa* L., *Haloxylon salicornicum*, and *Zygophyllum propinquum* were selected for their phytochemical analyses. The effect of soil and climatic conditions on the plant metabolites was investigated. Plant samples were collected at the beginning of March (winter) and the end of August (summer) separately to evaluate the effect of climatic conditions on plant components and their medicinal value. Soil samples were also collected for analysis to find any correlation between plant components and soil composition. Soil and plant samples were collected during the late winter and late summer of the same year. Quantitative analyses of soil samples showed differences in soil phosphorus, iron, magnesium, and as well as pH. These elements were higher in winter than in summer. On the other hand, nitrogen and electrical conductivity were higher in summer. However, there were no significant differences between summer and winter for calcium, potassium, sodium, bulk density, and soil water content. Physiological and biochemical analyses on the aerial parts of the selected plants showed significant differences in carbohydrate content between summer and winter. In fact, they were higher in winter for all the plants studied. Lipid content was higher in summer than in winter. The protein contents of *C. spinosa* L. were 14% higher in winter, while those of *H. salicornicum* were 21% higher in summer. *Z. propinquum* proved to be the most salt-tolerant plant, followed by *C. spinosa* L. and *H. salicornicum*. The alkaloid and saponin content of the plants was higher in summer than in winter. There was no significant difference between summer and winter in the levels of phenolic compounds and flavonoids in the plants studied. Based on these results, seasonal changes appear to significantly affect certain medicinal compounds, while other compounds remain relatively constant throughout the year.

## 1. Introduction

Wadi Al-Rummah is considered one of the prominent natural phenomena in the Kingdom of Saudi Arabia due to its length and width, which exceeds many other valleys in the region. Wadi Al-Rummah extends from latitude 26′00″85° and 26′40″36° N and longitude 44′20″49° and 44′00″57° E. Wadi Al-Rummah contains fluvial sediments [1]. This valley is rich in diverse species of medicinal plants. Up till 2023, around 59 plant species found at various sites in the different geomorphological landscapes of Wadi Al-Rumma, including 26 plants of medicinal importance, have been recorded [2,3].

Medicinal plants are the main source of drugs and active substances used in the pharmaceutical industry, and their importance is increasing with the progress of civilization and the growing need for medicine and the expansion of its uses [4]. Plant extracts contain numerous compounds, including organic acids, aromatic acids, coumarins, flavonoids, tannins, alkaloids, glycosides, terpenoids and steroids, and certain toxic gases [5].

Seasonal changes have a significant impact on the growth and chemical composition of plants. The main factor in seasonal changes is climate, and climatic conditions are one of the most important environmental factors affecting the composition and distribution of vegetation as they control the formation of plant communities by influencing the species that make up these communities; soil characteristics have an impact on the growth and distribution pattern of plants [6]. A study by Al-Qahtani [7] on four plant species collected in different localities in Saudi Arabia revealed significant differences in the chemical compositions of the plants studied, collected in different regions or the same area at different seasons. These differences can be attributed to environmental differences due to seasonal and spatial variations. Chaves et al. [8] examined seasonal variations in the chemical composition of two plants used in traditional Brazilian medicine, *Guapira graciliflora* and *Pseudobombax marginatum*. Their results showed significant seasonal variations in the chemical composition of extracts from the two plants, with certain concentrations of total flavonoids and total polyphenols differing significantly between the seasons studied.

The world’s most ancient civilizations were familiar with phytotherapy, knowing how to benefit from the therapeutic properties of certain plants, which were then harvested in different geographical areas [9]. Scientists used them to treat diseases by taking wild plants or parts of them in their natural state and applying them to the diseased part or organ. Humans have been searching for plants that reduce this pain since ancient times, which encourages today’s humans to take an interest in nature and discover more than what was previously known [10].

*C. spinosa* is one of the few shrubby species that offers many qualities and uses. As a spontaneous, xerophilous plant, it has an important ecological role, occupying soils on which few other plant species can survive. It tolerates the harsh climatic conditions of arid and semi-arid zones, as well as extreme temperatures [11]. *C. spinosa* contains several active ingredients, such as rutin glycosides, myrosinase, caproic acid, pectic acid, saponins, alkaloids, stachydrine, and flavonoids [12]. The chemical investigation of plants has led to the isolation of many biologically active compounds used today [13]. It is known in traditional medicine for its diuretic, astringent, tonic, and antirheumatic properties [14].

The family Zygophyllaceae contains approximately 27 genera and 285 species. The largest genus in this family is Zygophyllum with approximately 80 species. The study of these different species has revealed the presence of a large number of bioactive compounds belonging to different chemical classes, such as triterpenes, sterols, flavonoids, saponins, polyphenols, and essential oils [15]. A study aimed to characterize and document the wild plants of the Zygophyllaceae family in arid and semi-arid regions and documented important detailed information on medicinal plants of the Zygophyllaceae family; the data provided can be used, especially for the development of new drugs [16]. *Z. propinquum* is used in traditional medicine to treat conditions, such as diabetes, asthma, gout, rheumatism, and high blood pressure. These benefits are attributed to the bioactive compounds it contains [17].

Azhar et al. [18] has reported important information about the locally available *H. salicornicum* that may be useful for initiating various economic and healthcare programs in the region. This species can be used as a soil binder to preserve sandy soils and a source of poverty alleviation for local communities. The local people are well aware of their medicinal uses. Containing several alkaloids in the aerial parts, a piperidyl alkaloid, haloxynine, was isolated. Out of the 18 alkaloids identified, 10 were reported for the first time from this plant [19,20]. Fruits and vegetables containing flavonoids showed anticancer activity [21]. The presence of classes of phytochemicals, such as flavonoid, alkaloid, and tannin, showed cytotoxic effects [22].

The aim of this work is the phytochemical analysis and study of the impact of environmental factors on the concentration of these chemical constituents. In Saudi Arabia, and specifically, in the Al-Rummah Valley of Qassim, these plants are traditionally used to treat ailments like fever, headache, and cancer. Our objective is to select suitable times of the year and areas in the valley that result in the highest yield of these phytochemicals. The outcomes of this work may be useful in developing potent local low-cost and economic medicines against various diseases.

## 2. Materials and Methods

### 2.1. Study Areas

**Figure 1:**
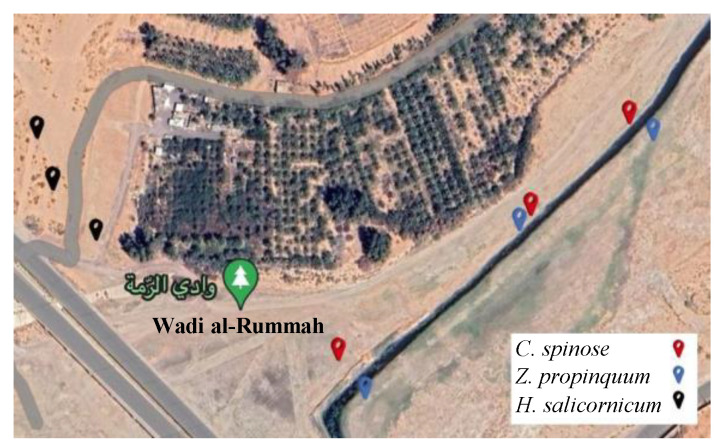
Locations of the study plants in Wadi Al-Rummah in Qassim, Saudi Arabia.

The study was carried out in the part of Wadi Al-Rummah located in Unaiza Governorate, Al-Qassim region, at latitude 26′1444009° N, longitude 43′95307451° E (Figure 1).

The study site and the plant species on which the study is based (three samples for each plant species and soil from under each sampled plant) were selected. Field trips were conducted during 2023 to collect plant and soil samples.

### 2.2. Soil Analysis

#### 2.2.1. Physical Analysis of Soil

The moisture content of soil. A fresh soil sample (50 g) was taken from each site near the plants under study. Roots and stones were removed and placed in a petri dish, placed in a drying oven at 105 °C for 4 h, cooled in a desiccator, and weighed; oven drying and weighing was repeated several times until the weight of the sample was stable [23]. The formula is as follows:

*Moisture percentage = (fresh soil weight − soil weight after drying)/soil weight after drying in the oven × 100*

Estimation of Soil Texture. Soil samples were analyzed for texture using the Hydrometer Method after the initial treatments. Hydrochloric acid HCL 0.1 N and water washing were used to remove dissolved salts and calcium carbonate, followed by hydrogen peroxide H_2_O_2_ 30% and washing with distilled water to expel organic matter. The sample was separated using Calgon (sodium hexametaphosphate) and then the percentage of sand, silt, and clay was calculated to Day’s method [24].

#### 2.2.2. Chemical Analysis of Soil

Determination of bulk density and hydraulic properties of soil. Bulk density (BD) was estimated in the field using a cylinder of known size, 5 cm in height and 5.25 cm in internal diameter, and dried at 1.5 °C until weight stabilization [25], and was calculated from the following relationship:

*BD* = *Ms*
*/V*

*Ms* is the weight of the dry soil, *V* is the volume of the cylinder.

True density was estimated by the soil conservation method [25].

Determination of soil pH. The air-dried soil was prepared from under the plants under study at depths of 0–30 cm, 30–60 cm, and 60–100 cm separately and passed through a 2 mm sieve to prepare the soil extract at a ratio of 1:5 (1 g soil to 5 mL distilled water). The pH meter was set using solutions with known pH values. The pH value of each soil solution was then recorded by the pH meter [26].

Determination of soil nutrients. This was performed by preparing soil samples for testing with a Flame Photometer and an Atomic Spectrometer [27].

Nitrogen Determination (N). N was determined by the Caldal method and included three main steps: digestion, distillation, and titration. This was conducted on all soil samples under study at depths of 0–30, 30–60, and 60–100 cm [28].

Phosphorous determination (P). It was estimated by colorimetric method (Spectrophotometer) using the molybdenum blue method [29].

### 2.3. Plant Sampling and Growth Measurements

**Figure 2:**
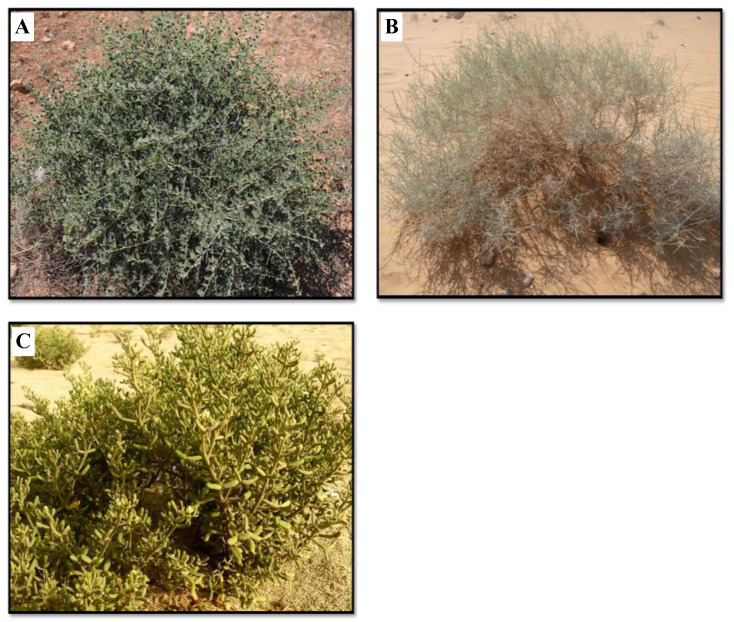
Medicinal plants chosen to carry out the study. (**A**) *C. spinosa* L., (**B**) *H. salicornicum*, and (**C**) *Z. propinquum*.

Three plant species were studied: *C. spinosa* L., *Z. propinquum*, and *H. salicornicum* (Figure 2). Plant species were identified under the guidelines of the Ministry of Agriculture and water, Saudi Arabia [30].

The samples were collected from three sites in Wadi Al-Rummah, Al-Qassim over two different seasons (winter, beginning of March 2023, and summer, end of August 2023) to examine the effect of seasonal changes on the chemical content. The plant samples were collected completely from the specified sites and thoroughly cleaned of soil and other plant materials so that these materials do not affect the validity of the obtained results. After that, the air-drying phase was started by leaving the plant samples on filter papers in a shaded and well-ventilated area at a moderate temperature. They were stirred daily so that the substrate was not exposed to mold.

The plants were left to dry for two weeks. Then the milling process was carried out. The dried plant samples were ground into a fine powder and the powder samples were kept in glass containers for analysis.

### 2.4. Plant Analysis

#### 2.4.1. Extraction and Quantification of Shoot Cations

A total of 0.5 g of powdered dry plant samples were transferred to a glass test tube and 10 mL of HNO_3_ nitric acid was added and heated on a hotplate until complete digestion was reached. A water distiller was then used to transfer the completely digested mixture from the tube to a volumetric flask by filtering through filter paper (110 mm diameter) with a pore diameter of 20–25 μm and the total volume was completed to 100 mL of distilled water. Measurements were made with a Flame Photometer and an Atomic Spectrometer. The plant contents of Fe, Cu, Zn, Mn, N, P, K, Ca, Mg, and Na were measured according to Soltanpour and Schwab [31]. Each sample was repeated three times, and the average was calculated.

#### 2.4.2. Carbohydrate Content Determination

The method described by Anthrone [32] was followed to estimate the carbohydrate content of plant samples. The light absorbance of the samples was read at 630 nm using a DR 6000™ UV VIS Spectrophotometer (Thomas Scientific, Denver, CO, USA). Total carbohydrates were calculated as a percentage of glucose present in the sample extract using the following equation:

*Carbohydrates (%) = 25 × A*1*W* × *A*2 × *100*

*A*1: Absorbance of the sample at 630 nm

*A*2: Absorbance standard at 630 nm

*W*: Weight of extracted sample (g)

#### 2.4.3. Determination of Total Plant Ash

The gravimetric method was followed by furnace burning, as described by James [32], to determine the total Ash content in the plant samples. This method is based on estimating the loss of organic matter by taking a specific weight of the plant samples and using a well-known weight (*W*1) to burn 5 g of the dried plant powder sample in an oven at 550 °C until complete Ash is reached. The dried plant powder sample was left to cool in a desiccator and then weighed (*W*2). The following equation was used:

*Ash (%) = (W*1 *− W*2*)/Weight of sample × 100*

#### 2.4.4. Crude Fat Determination

The non-polar solvent extraction method, as reported by Kirk and Sawyer [33], was used to determine the crude fat content in the plant samples using the following formula:

*Total Fat (%) = 100 × precipitate weight/plant weight*

#### 2.4.5. Nitrogen Determination (N)

Nitrogen in the samples is determined by the Caldal method [29] and includes three main steps: digestion, distillation, and titration.

#### 2.4.6. Extraction and Estimation of Crude Protein

Crude protein content was measured by the Kjeldahl method based on the total N content in the sample according to AOAC [34], and was determined using the following formula:

*Protein percentage (%) = Nitrogen percentage × 6.25*

#### 2.4.7. The Analyses of Compounds from the Extracts of the Plants Selected in the Study

This was performed by high-precision liquid chromatography (HPLC) spectrophotometric absorption according to the method of Harborne and Grayer [35]. HPLC analysis was carried out using an Agilent 1260 series. The separation was carried out using Zorbax Eclipse Plus C8 column (4.6 mm × 250 mm i.d., 5 μm). The mobile phase consisted of water (A) and 0.05% trifluoroacetic acid in acetonitrile (B) at a flow rate 0.9 mL/min. The mobile phase was programmed consecutively in a linear gradient as follows: 0 min (82% A); 0–1 min (82% A); 1–11 min (75% A); 11–18 min (60% A); 18–22 min (82% A); 22–24 min (82% A). The multi-wavelength detector was monitored at 280 nm. The injection volume was 5 μL for each of the sample solutions. The column temperature was maintained at 40 °C.

#### 2.4.8. Analysis of Total Alkaloid

To determine the total alkaloids, the method of Shamsa et al. [36] was used. The plant materials (5 g) were ground and extracted with methanol for 24 h in a continuous extraction apparatus at 260 °C. The extract was filtered, and methanol was removed on a rotary evaporator under a vacuum at a temperature of 450 °C to dry. A part of this residue was dissolved in HCl 2N and then filtered. One mL of this solution was transferred to a separate funnel and washed with 10 mL chloroform (3 times). The pH of this solution was adjusted to be neutral with NaOH 0.1 N. Then 5 mL of BCG solution and 5 mL phosphate buffer were added to this solution. The mixture was shaken and the complex formed was extracted with 1, 2, 3, and 4 mL chloroform by vigorous shaking. The extracts were collected in a 10 mL volumetric flask and diluted to volume with chloroform. The absorbance of the complex in chloroform was measured at 470 nm.

#### 2.4.9. Analysis of Total Tannins

The tannin contents were determined by the method of Broadhurst and Jones [37] by using Tannic as a reference compound. A volume of 400 µL of extract was added to 3 mL of a solution of vanillin (4% in methanol) and 1.5 mL of concentrated hydrochloric acid. After 15 min of incubation, the absorbance was read at 500 nm.

#### 2.4.10. Analysis of Total Saponins

One gram of powder was mixed with 30 mL of ethanol in a 100 mL conical flask. The suspension was left standing for 30 min pre-leaching at ambient temperature. The suspension was then rapidly cooled to ambient temperature in an iced water bath and filtered through a 0.45 µm membrane filter. The clear extract was collected and its TSC was determined. All tubes were placed in a water bath set at 65 °C until the methanol was evaporated to dryness (~5 min). Then, 0.5 mL of 4% vanillin in ethanol (*w*/*v*) was added to each tube followed by 2.5 mL of 72% H_2_SO_4_ (*v*/*v*). The tubes were covered, vortexed, incubated in a water bath at 60 °C for 15 min, and then cooled for 5 min in water at ambient temperature. The absorbance of the solutions was measured at 560 nm using a Biosystem 310 spectrophotometer after zeroing with the blank. The absorbance values obtained were plotted against the concentrations to construct a standard curve.

### 2.5. Statistical Analysis

Principal components between plant species samples were analyzed using Minitap statistical analysis software.12, and correlations between plant species and seasons (winter and summer) were examined using SPSS.12 and ANOVA analysis of variance.

## 3. Results

### 3.1. Seasonal Variation in Soil Properties

**Figure 3:**
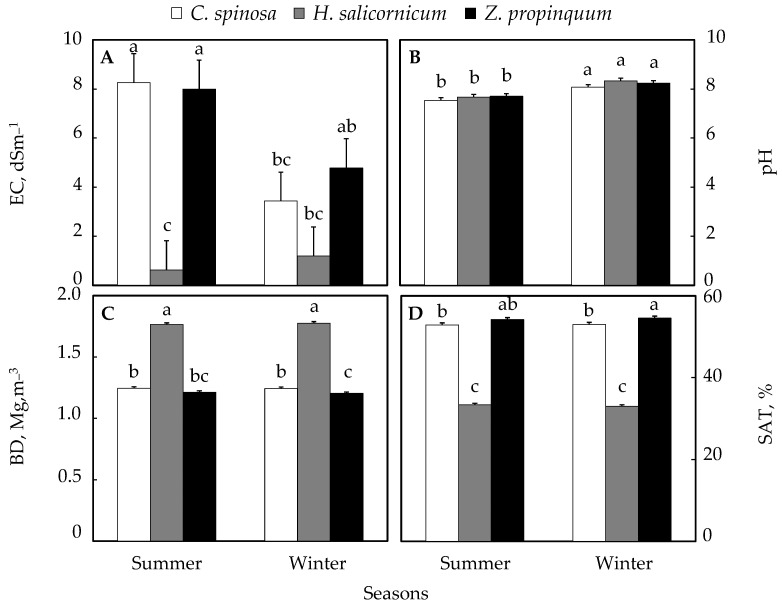
Soil proprieties under the selected plants according to season. (**A**) Electrical conductivity (EC), (**B**) pH, (**C**) Bulk density (BD), (**D**) Moisture content (SAT). Values are means of triplicates. SE presented as a vertical bar above each mean. The same letters indicate no significant difference regarding the ANOVA analysis of variance.

The variations in soil properties according to the season are illustrated in Figure 3. For the electrical conductivity (EC) parameter, Figure 3A showed significant differences in salts in the soil under *C. spinosa* between winter and summer, where it appeared that in summer, it was higher than in winter, by 141%; in the soil under *Z. propinquum*, it appeared that in summer, it was higher than in winter, by 67%, while for *H. salicornicum*, it was higher in winter than in summer by 90% (Figure 3A).

The pH in winter is higher than in summer in all plant soils, and the percentage of increase in the soil of *C. spinosa* was 7%; the soil of *Z. propinquum* showed that it was higher in winter than in summer by 7%, while for *H. salicornicum*, the increase in winter was 9% higher than in summer (Figure 3B).

The bulk density of soil (BD) was very similar between winter and summer in the soils of all plants, where it increased in summer from winter by 0.3% in the soil of *C. spinosa*, 0.8% in the soil of *Z. propinquum*, and 0.6% in the soil of *H. salicornicum* in winter from summer (Figure 3C).

As for moisture content, it was also very similar between winter and summer in the soils of all plants, as it increased in winter from summer by 0.2% in the soil of *C. spinosa*, 0.7% in the soil of *Z. propinquum*, and 1% in the soil of *H. salicornicum*, while the soil of *H. salicornicum* increased in summer from winter (Figure 3D).

Analysis of soil variability with season (summer–winter) and plant species showed that for electrical conductivity, there were no significant differences between the different species, but there were significant differences between the same plant species and the summer–winter seasons (Table S1).

As for pH and alkalinity, there were significant differences between the summer and winter seasons regardless of the plant species (Table S1).

For salts, bulk density, and moisture content, there were no significant differences for the same plant species between the summer and winter seasons (Table S1).

### 3.2. Seasonal Variation in Soil Nutrient Content

**Figure 4:**
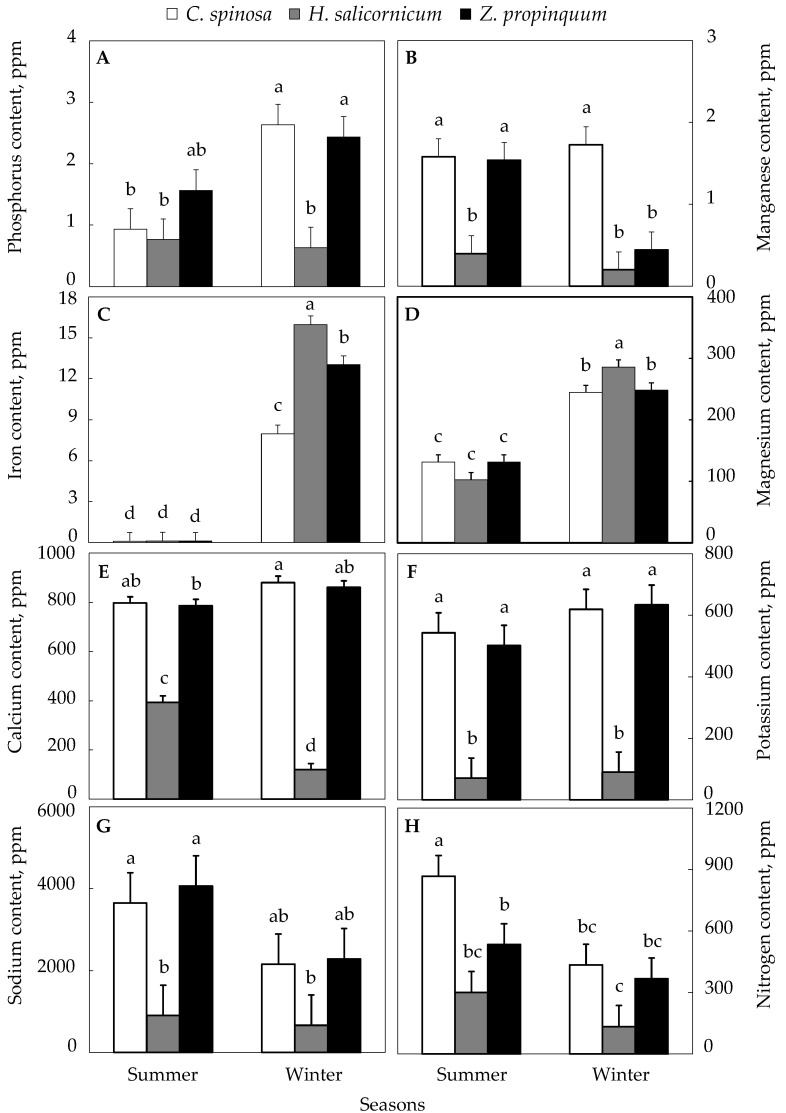
Soil nutrients under the selected plants according to season. (**A**) Phosphorus content, (**B**) Manganese content, (**C**) Iron content, (**D**) Magnesium content, (**E**) Calcium content, (**F**) Potassium content, (**G**) Sodium content, (**H**) Nitrogen content. Values are means of triplicates. SE presented as a vertical bar above each mean. The same letters indicate no significant difference regarding the ANOVA analysis of variance.

Figure 4A showed significant differences in the phosphorus content of the soil under the *C. spinosa* plant between winter and summer. In winter, it was 2.63 ppm and in summer, it was found to be 0.93 ppm. It appeared to be higher in winter than in summer with a percentage increase of 182%. In the case of *Z. propinquum,* the soil phosphorous contents were 2.43 ppm in winter and 1.57 ppm in summer. It was higher in winter than in summer by 55%, while for *H. salicornicum,* the phosphorus content of the soil in winter was 0.63 ppm and in summer, 0.77 ppm. It was higher in the summer than winter by 21%.

There were also significant differences in the manganese content of the soil under *C. spinosa* between winter and summer as shown in Figure 4B. In winter, it was 1.73 ppm, and in summer, it was 1.58 ppm, where it appeared to be higher in winter than in summer by 9%. Soil analysis of land under *Z. propinquum* showed 0.44 ppm Mn in winter and 1.54 ppm in summer, which was 247% higher in summer than in winter. A reverse trend was found in soil manganese under *H. salicornicum,* showing a 99% higher value in summer (0.77 ppm) than in winter (0.63 ppm).

As for the iron content of the soil, it was higher in winter than in summer in all plant soils. This content was ~72 times, ~120 times, and ~130 times higher in winter compared to summer in *C. spinosa*, *Z. propinquum,* and *H. salicornicum*, respectively (Figure 4C).

The magnesium content of the soil in winter is higher than in summer in all plant soils, and the percentage of increase in the soil of *C. spinosa* was 86%; the soil of *Z. propinquum* showed that it was higher in winter than in summer by 89%, while for *H. salicornicum*, Mg content was ~3 times higher in winter compared to summer (Figure 4D).

It also turned out that there were significant differences in the calcium content of the soil under *C. spinosa* between winter and summer. In winter, it was 10% higher than in summer, and the soil of *Z. propinquum* was 10% higher than in summer. For *H. salicornicum*, the increase in soil calcium content in summer was ~3 times higher than in winter (Figure 4E).

As for the potassium content of the soil, it turned out that there were significant differences in the soil of *C. spinosa* between winter and summer, as it appeared that in winter, it was higher than in summer by 14%; the soil of *Z. propinquum* was higher than in summer by 26%; and the increase in potassium content in the soil of *H. salicornicum* in winter was higher than summer by 27% (Figure 4F).

The sodium content of the soil in summer was higher than in winter for all the plants; the percentage increase for *C. spinosa* was 69%, while the soil of *Z. propinquum* showed that in winter, it was higher than in summer by 78%, while for *H. salicornicum*, the increase in winter compared with summer was 36% (Figure 4G).

It was also clear that the nitrogen content of the soil in summer is higher than in winter in all plants, the percentage of increase in the soil of *C. spinosa* was 100%, and the soil of *Z. propinquum* showed that it was higher in winter than in summer by 45%, while for *H. salicornicum*, the increase in winter compared to summer was 125% (Figure 4G).

Analysis of soil variability under plant species with season (summer–winter) and plant species showed that there were no significant differences in soil phosphorus and potassium content under *C. spinosa* and *Z. propinquum* in winter, while significant differences appeared between them and the soil under *H. salicornicum* (Table S1). In the summer season, there were no significant differences in all soils under the plants (Table S1).

As for manganese, there were no significant differences in the soil under *Z. propinquum* and *H. salicornicum* in winter, while there were significant differences between them and the soil under *C. spinosa*. In the summer season, there were no significant differences in the soil under *C. spinosa* and *Z. propinquum*, while there were significant differences between them and the soil under *H. salicornicum* (Table S1).

Iron showed significant differences in winter in the soil under *H. salicornicum*, followed by *Z. propinquum* and *C. spinosa*. There were no significant differences in all the soils under the plants in summer (Table S1).

Soil magnesium content did not show significant differences in soils under *C. spinosa* and *Z. propinquum* in winter, while significant differences appeared between them and the soil under *H. salicornicum* and were higher in the soil under *Z. propinquum*. In the summer season, there were no significant differences in all soils under the plants (Table S1).

The calcium content of the soil showed significant differences in all soils under the plants and in different seasons (Table S1).

There were no significant differences in soil sodium content in the winter season for all plant species, as well as between the soil under *C. spinosa* and *Z. propinquum* in summer, while there were significant differences between them and the soil under *H. salicornicum* in the summer season (Table S1).

Soil nitrogen content did not show significant differences in soils under *C. spinosa* and *Z. propinquum* in winter and *H. salicornicum* in summer, while there were significant differences between soils under *C. spinosa* and *Z. propinquum* in summer and *H. salicornicum* in winter (Table S1).

### 3.3. Seasonal Variation in Primary Metabolites

**Figure 5:**
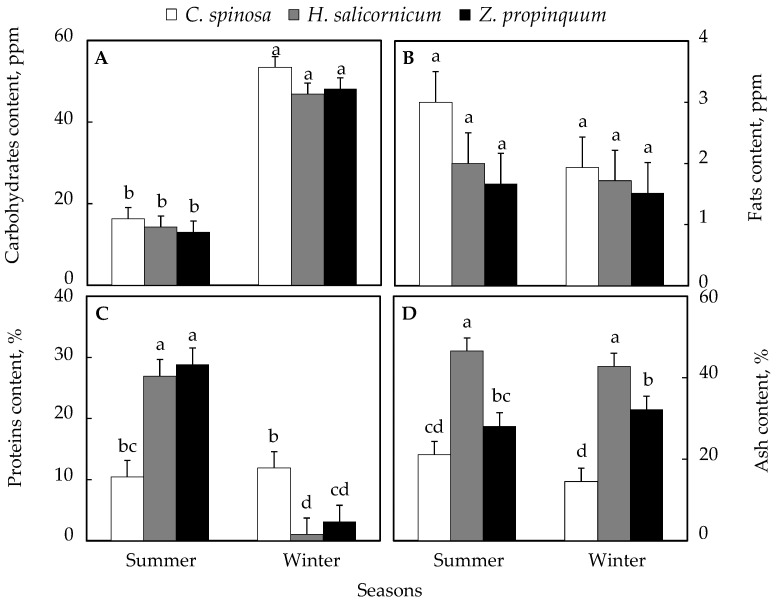
Seasonal variation in primary metabolites of selected plants. (**A**) Carbohydrate content, (**B**) Fats content, (**C**) Proteins content, (**D**) Ash content. Values are means of triplicates. SE presented as a vertical bar above each mean. The same letters indicate no significant difference regarding the ANOVA analysis of variance.

Figure 5A shows that there were significant differences in the carbohydrate content in the plants, as it was higher in winter than in summer for all plants. The percentage increase in *C. spinosa* plants was 227%. Similarly, *Z. propinquum* and *H. salicornicum* showed that these contents were higher in winter than summer. The increase was 269% and 229%, respectively.

Lipid contents in the various plants were higher in summer than in winter. The percentage increase in *C. spinosa*, *Z. propinquum,* and *H. salicornicum* was 55.4%, 10.6%, and 16.3%, respectively (Figure 5B).

Plant protein concentration was found to be slightly higher in winter than in summer in *C. spinosa*, with a percentage of 14% compared with summer (no significant difference), while in *Z. propinquum* and *H. salicornicum*, the increase was ~9 times and ~26 times, respectively, in summer compared to winter (Figure 5C).

Ash content was higher in winter than in summer in *C. spinosa* and *Z. propinquum*. The increase was 44.5% and 14.3%. In *H. salicornicum*, Ash content was 8.8% higher in summer than in winter (Figure 5D).

### 3.4. Seasonal Variation in Plant Nutrient Content

#### 3.4.1. Plant Macronutrients

**Figure 6:**
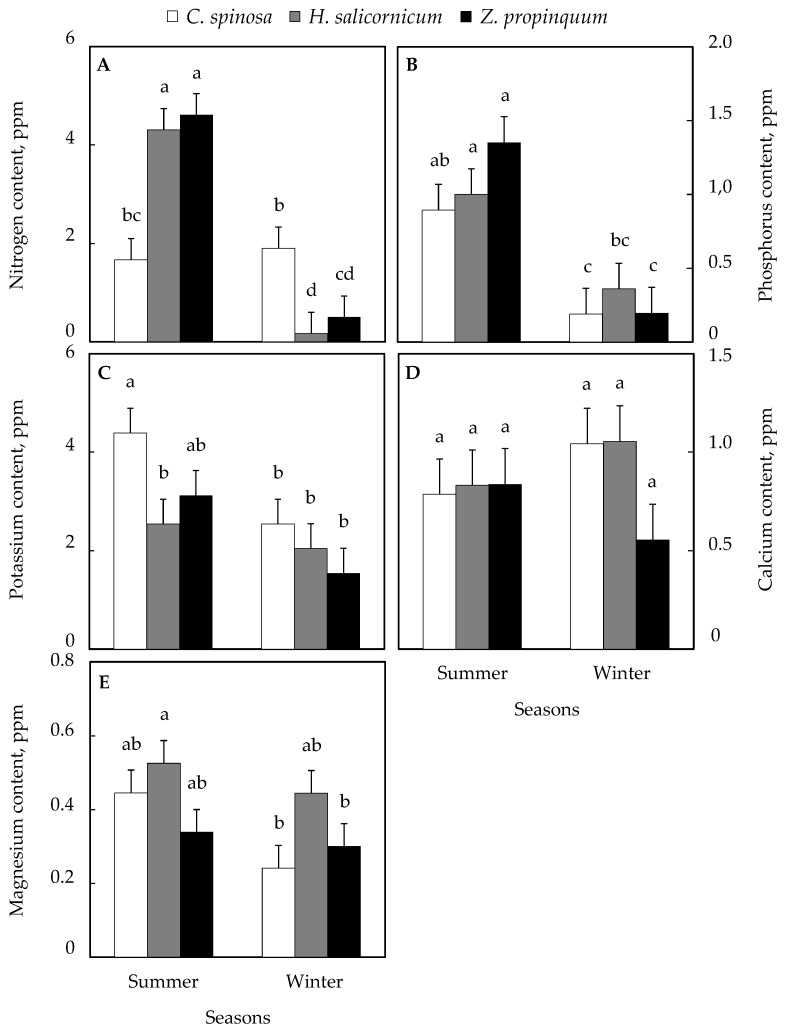
Seasonal variation in macronutrient contents of selected plants. (**A**) Nitrogen content, (**B**) Phosphorus content, (**C**) Potassium content, (**D**) Calcium content, (**E**) Magnesium content. Values are means of triplicates. SE presented as a vertical bar above each mean. The same letters indicate no significant difference regarding the ANOVA analysis of variance.

The nitrogen content in various plants was revealed to be higher in winter than in summer in *C. spinosa* and the increase was 13.8%, while *Z. propinquum* and *H. salicornicum* showed that in summer, it was ~9 times and ~26 times higher than in winter, respectively (Figure 6A).

Phosphorus contents were higher in summer than in winter for all plants and the rate of increase for *C. spinosa*, *Z. propinquum,* and *H. salicornicum* was ~5 times, ~7 times, and ~3 times, respectively (Figure 6B).

Similarly, all plants had higher potassium contents in summer than in winter. The increase was 73% in *C. spinosa*, 103% in *Z. propinquum,* and 20% in *H. salicornicum* (Figure 6C).

Calcium contents were higher in winter than in summer in *C. spinosa* (32% increase) and *H. salicornicum* (27% increase), while *Z. propinquum* showed that these contents were 51% higher in summer than in winter (Figure 6D).

Magnesium content was higher in summer than in winter in all plants with an 84% increase in *C. spinosa*, 13% in *Z. propinquum,* and 18% in *H. salicornicum* (Figure 6E).

#### 3.4.2. Plant Micronutrients

**Figure 7:**
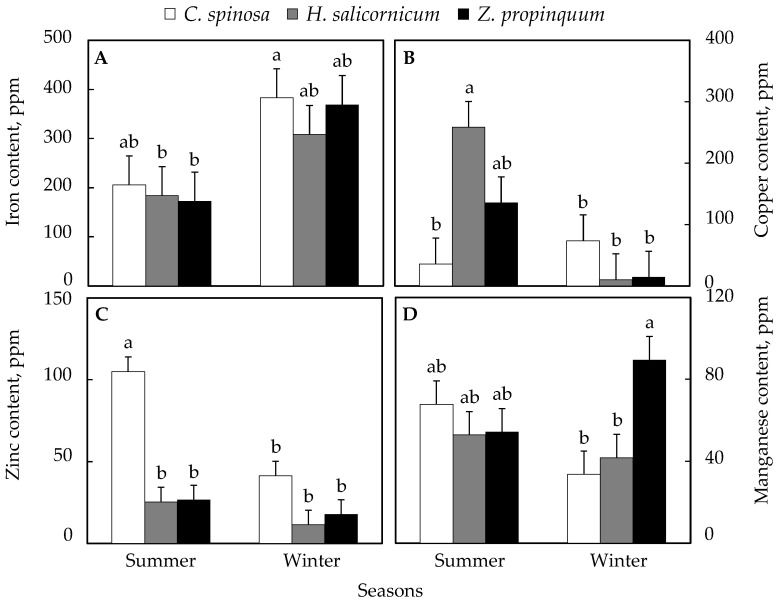
Seasonal variation in micronutrient contents of selected plants. (**A**) Iron content, (**B**) Copper content, (**C**) Zinc content, (**D**) Manganese content. Values are means of triplicates. SE presented as a vertical bar above each mean. The same letters indicate no significant difference regarding the ANOVA analysis of variance.

Iron content was higher in winter than in summer in all plants. *C. spinosa* increased by 86%, 113% in *Z. propinquum*, and 68% in *H. salicornicum* (Figure 7A).

Copper concentration was higher in winter than in summer in *C. spinosa* (104% increase), while in *Z. propinquum* and *H. salicornicum*, these levels were ~10 times and ~26 times higher in summer than in winter, respectively (Figure 7B).

Zinc levels were higher in all plants in summer than in winter. The increase was 154%, 50%, and 123% in *C. spinosa*, *Z. propinquum,* and *H. salicornicum*, respectively (Figure 7C).

Manganese concentration was higher in summer than in winter in *C. spinosa* (102%) and *H. salicornicum* (27%). The contrary was observed in *Z. propinquum*, in which values were 65% higher in winter than in summer (Figure 7D).

### 3.5. Seasonal Variation in Secondary Metabolites

**Figure 8:**
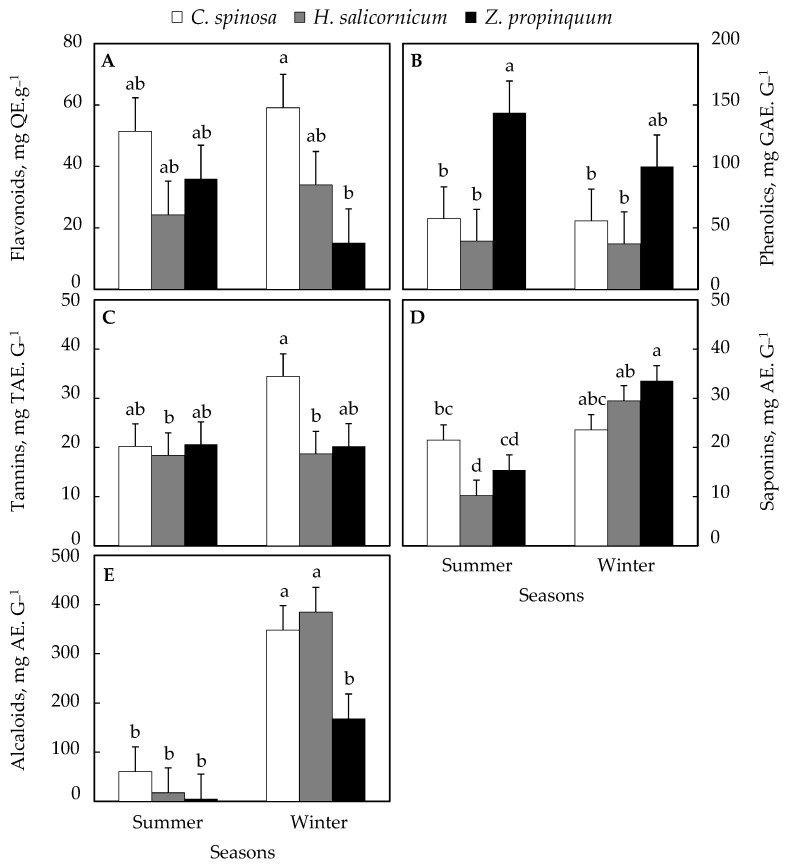
Seasonal variation in secondary metabolites of selected plants. (**A**) Flavonoids content, (**B**) Phenolics content, (**C**) Tannins content, (**D**) Saponins content, (**E**) Alcaloids content. Values are means of triplicates. SE presented as a vertical bar above each mean. The same letters indicate no significant difference regarding the ANOVA analysis of variance.

The total plant concentration of flavonoids was higher in winter than in summer in *C. spinosa* and the increase was 14.8%, while *Z. propinquum* showed that it was higher in summer than in winter by 136.6% and *H. salicornicum* in winter than in summer by 39.9% (Figure 8A).

In all plants, the concentration of phenolic compounds in shoots was higher in summer than in winter. Increases of 3%, 44%, and 5% were recorded in *C. spinosa*, *Z. propinquum,* and *H. salicornicum*, respectively (Figure 8B).

Regarding tannin concentration, it was higher in winter than in summer in *C. spinosa* and *H. salicornicum* (71% and 2%, respectively), while in *Z. propinquum*, it was higher in summer than in winter by 2% (Figure 8C).

As for the concentration of saponins, it was higher in winter than in summer in all plants, and the percentage increase in *C. spinosa*, *Z. propinquum,* and *H. salicornicum* was 10%, 118%, and 189%, respectively (Figure 8D).

The concentration of alkaloids in shoots was ~6 times, ~32 times, and ~22 times higher in winter than in summer in *C. spinosa*, *Z. propinquum,* and *H. salicornicum*, respectively (Figure 8E).

### 3.6. Analysis of Variance Between Plant Species and Seasons (Summer and Winter)

The analysis of variance for statistically significant plants in primary and secondary metabolites and in nutrient analyses with seasonal fluctuations (summer and winter) and plant types showed significant differences in the plant content of protein, nitrogen, copper, zinc, manganese, and saponin by plant type and season (Table S2).

Protein and nitrogen were higher in summer than in winter and the pyramid plant record *Z. propinquum* and *H. salicornicum* recorded the highest rate in the summer season. Cu was higher in *H. salicornicum* in summer than in winter and in the rest of the plant species in both seasons. Zinc in *C. spinosa* was higher in summer than winter and higher than the rest of the plant species in both seasons. Manganese and saponin were higher in *Z. propinquum* in winter than in summer and the rest of the plant species in both seasons (Table S2).

There were no significant differences in carbohydrate, fat, Ash, phosphorus, potassium, calcium, magnesium, iron, flavonoids, phenolic, tannins, and alkaloid content by plant type and season (Table S2).

### 3.7. Principal Component Analysis (PCA) of Soil Properties and Nutrients

Principal component analysis (PCA) is an important dimensionality reduction technique, as it significantly reduces the number of characteristics in a dataset without losing too much information, making it easier to analyze and visualize. In addition to extracting meaningful features from raw data, principal component analysis can be used to visualize data. Principal component analysis can be used to visualize high-dimensional data in a lower-dimensional space, which facilitates understanding relationships and patterns.

Therefore, the KMO (Kaiser–Meyer–Olkin) test was performed and found to be 0.625, which is greater than 0.5, and thus allows principal component analysis to be performed on the components obtained.

The number of principal components of variance after applying statistical analysis by calculating the Eigenvalues using PCA was five. Still, only two principal components of variance (PC1 and PC2) were used as they accounted for 90% of the cumulative variance. PC1 explained 61.48% of the variance, while PC2 explained 28.72%.

**Figure 9:**
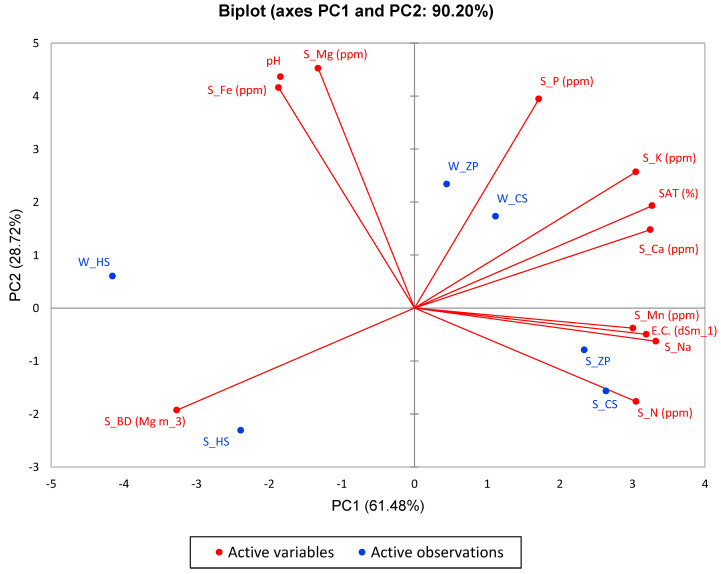
Principle component analysis (PCA) for soil proprieties and nutrients. Distribution of the studied characteristics by Biplot method.

Referring to Figure 9 and Table S3, we can see that the soil properties represented by the PC1 component were sodium 0.88, bulk density 0.85, saturation 0.85, calcium 0.84, electrical conductivity 0.81, nitrogen 0.74, potassium 0.74, and manganese 0.72.

The soil properties of the PC2 component were magnesium 0.76, soil pH 0.71, iron 0.64, and phosphorus 0.58.

### 3.8. Principal Component Analysis (PCA) of Plant Characteristics

The number of principal components of variance after applying statistical analysis by calculating Eigenvalues using PCA was five. Still, only four principal components of variance (PC1, PC2, PC3, and PC4) were used, accounting for 95.8% of the cumulative variance. PC1 explains 23% of the variance, PC2 accounts for 65.6%, PC3 accounts for 16.6%, and PC4 accounts for 13.6%.

**Figure 10:**
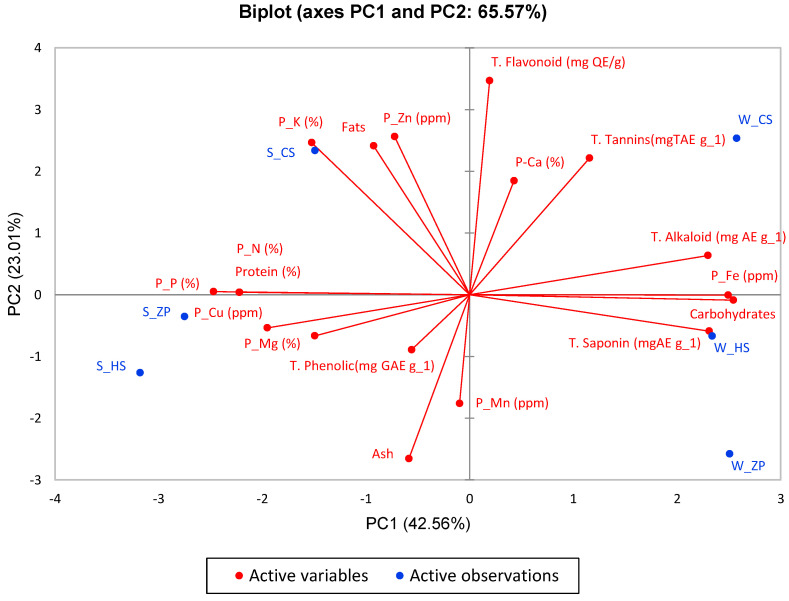
Principle component analysis (PCA) for plant characteristics. Distribution of the studied characteristics by Biplot method.

Referring to Figure 10 and Table S4, the plant characteristics represented by PC1 were as follows: carbohydrates with 0.97, iron with 0.93, phosphorus with 0.91, saponin with 0.8, alkaloid with 0.79, nitrogen with 0.74, protein with 0.74, protein with 0.74, and copper with 0.57.

Component PC2 found that the plant characteristics represented were as follows: flavonoids of 0.97, Ash of 0.57, zinc of 0.53, potassium of 0.49, lipids of 0.47, and tannins of 0.4.

PC3 was found to have plant characteristics of manganese with a value of 0.69 and calcium with a value of 0.52.

PC4 was found to have plant characteristics of phenolic phenols with a value of 0.69 and magnesium with a value of 0.63.

## 4. Discussion

The results show that soil properties change significantly between summer and winter. This may reflect plants’ adaptation strategies to seasonal changes.

*Z. propinquum* seems to be the most tolerant of salinity, followed by *C. spinosa*, while *H. salicornicum* seems to prefer less saline soils.

The results showed an increase in iron content during winter. This was consistent with a study conducted by Zhang et al. [38] in dry subtropical regions, where soil iron availability increases during the rainy seasons. This was consistent with a study by Chen et al. [39] that focused on seasonal changes in iron levels in Zhanjiang Bay in China and showed that iron levels differed significantly between seasons, rising in autumn due to rainfall and river inputs.

The higher electrical conductivity in summer compared to winter was consistent with the findings of Al-Busaidi and Cookson [40] in their study of coastal soils in Oman. This is usually attributed to increased evaporation and the accumulation of salts in the surface layer of the soil during summer.

The study showed that the pH was higher in winter, 8.2 compared to 7.6 in summer. These results reflect seasonal effects on soil chemical properties. This may be due to the increased moisture in winter leading to washing away some of the acidic ions from the soil. Rain washes the soil and removes acidic ions, such as hydrogen, iron, and aluminum, from the surface layer of the soil. This leaching reduces the concentration of acidic ions that would normally be responsible for lowering the pH, leading to increased alkalinity. This process is known as ‘alkaline leaching’ and is more common in sandy and desert soils that experience rapid water infiltration.

These results are consistent with the study of Li et al. [41], which showed that pH increased in winter in deserts and semi-arid areas due to reduced microbial activity and increased leaching by rainfall. Maselesele et al. [42] also showed that sandy and arid soils exhibit large fluctuations in pH between seasons due to rapid leaching processes.

The results showed that the nitrogen concentration in the soil was higher in summer (567 ppm) than in winter (311 ppm). Nitrogen is an essential nutrient that plants require in large quantities, as it is involved in the synthesis of amino acids, which are the building blocks of proteins, and in the construction of chlorophyll, which contributes to photosynthesis [43]. The high concentration of nitrogen in the soil during the summer is due to the increased activity of nitrogen-fixing bacteria at high temperatures. These bacteria play a crucial role in fixing atmospheric nitrogen and converting it into forms that can be absorbed and used by plants [44]. As temperatures rise, the activity of these microorganisms increases, thus increasing the amount of nitrogen available to plants in the soil. Biological nitrogen fixation processes require favorable temperature and are, therefore, more active during summer periods. In addition, the decomposition of organic matter in the soil increases during the summer, as higher temperatures accelerate the activity of microorganisms that decompose plant debris and other organic matter [45]. As these materials decompose, nitrogen is released as ammonium (NH_4_^+^) or nitrate (NO_3_^−^), which can be easily absorbed by plants. This rapid decomposition in summer contributes to a significantly higher availability of nitrogen in the soil compared to winter. These results are consistent with the study of Abdullah et al. [46], which showed that seasonal changes significantly affect the nitrogen cycle in soils. In arid and semi-arid environments, the activity of microorganisms is observed to increase in summer, leading to increased nitrogen fixation and the decomposition of organic matter.

The study showed that soil magnesium levels were higher in winter (260 ppm) compared to summer (122 ppm). Magnesium is an important nutrient involved in the formation of chlorophyll and plays an essential role in photosynthesis [47]. The difference in magnesium concentration between summer and winter can be explained by several factors. In winter, plant growth rates are significantly reduced, and plants are in a semi-dormant state, which means their need for nutrients, such as magnesium, is reduced. This leads to an accumulation of magnesium in the soil because it is not absorbed by plants as much as in summer, when plants are in an active growth state and need higher amounts of magnesium to support their vital processes. Increased moisture in winter also contributes to the solubilization of more magnesium compounds in the soil. These results are consistent with the study of Rezig et al. [48], which showed that high humidity in winter contributes to increasing the availability of magnesium in the soil by solubilizing insoluble compounds. It also showed that low plant growth reduces competition for nutrients, such as magnesium, leading to its accumulation in the soil.

Differences in soil properties between plant species suggest different strategies for coping with environmental stress [49]. The pattern of seasonal changes in nutrient availability, especially nitrogen and phosphorus, is in agreement with the results of Abdullah et al. [46] in their study of seasonal nutrient dynamics in desert ecosystems.

Significant differences in soil properties between summer and winter seasons were observed. This may lead to changes in the concentration and composition of active compounds in medicinal plants. Also, the timing of collecting medicinal plants has a significant impact on the concentration of their medicinal components. These results support the research of Christodoulou et al. [50], suggesting that the concentration of active substances in *C. spinosa* varied significantly between dry and rainy seasons. Analyses of the plant species, such as *C. spinosa*, *Z. propinquum*, and *H. salicornicum*, grown in soils with different physical and chemical properties showed a variety of medicinal compounds. Indeed, each species can produce new secondary compounds in response to environmental change. Studies by Amtaghri and Eddouks [51] discovered unique anticancer compounds in *H. salicornicum* created when these plants were exposed to a harsh desert environment.

Differences in soil properties affect the distribution and density of different plant species and the development of strategies to preserve the natural habitats of medicinal plants and develop sustainable methods for their collection [52,53]. Overall, this study showed that soil properties change significantly between summer and winter, highlighting the ability of desert plants to adopt sophisticated adaptation strategies to cope with these seasonal changes. For example, higher humidity in winter increases the availability of certain elements, such as magnesium and phosphorus, while summer heat increases the concentration of salts and minerals in the soil. This adaptation to climatic factors reflects the ability of desert plants to adapt to environmental changes. In addition, the ability of plants to absorb nutrients varies depending on seasonal changes. Indeed, low plant activity during winter leads to the accumulation of some elements, such as magnesium, in the soil due to lack of absorption, which enhances their utilization during the summer when plants need these elements to support rapid growth. The results show that each type of plant affects the surrounding soil differently, demonstrating that each plant follows a unique adaptation strategy to utilize available nutrients and adapt to environmental conditions.

*C. spinosa* showed an exceptional ability to extract manganese from the soil, indicating its effective strategy for adapting to soils with varying average properties. *C. spinosa* is a resilient plant capable of growing in diverse environments, thriving in soils with varying mineral and nutrient content, making it suitable for various environmental conditions. A study by Sabreena et al. [54] showed the important role of superabsorbent plants, such as *C. spinosa*, in phytoremediation. These plants have a high capacity to absorb heavy metals, especially manganese, from contaminated soils. A study also showed that *C. spinosa* adapts very well to soils with high concentrations of heavy metals, thus making it an ideal ecological choice for desert areas [55].

*Z. propinquum* seems to specialize in growing in saline environments. This plant shows high adaptability to soils with high concentrations of sodium and salts, as it has mechanisms that allow it to tolerate high salinity and retain moisture in the surrounding soil. These strategies help it grow in challenging environmental conditions, such as coastal soils and arid environments characterized by high salinity [56]. Plants, such as *Z. propinquum*, are of great importance in salt tolerance research, as they can contribute to the development of more salt-tolerant farming systems, which are vital in arid and desert regions that suffer from soil salinity. High levels of sodium and electrical conductivity, especially under *Z. propinquum*, salt stress may increase the production of some secondary compounds in medicinal plants as a defense mechanism. Thus, increasing the concentration of active compounds may enhance the medicinal properties of plants, as the study of Shomali et al. [57] showed that salt stress increases the production of flavonoids in some desert medicinal plants.

This study showed that the levels of most nutrients in the soil under *H. salicornicum* were low, except for iron, confirming the findings of Aloud et al. [58] in their theory on plant adaptation strategies. Differences in soil properties between plant species suggest different strategies to cope with environmental stress in desert environments. The high bulk density and low moisture content in the soil under *H. salicornicum* agree with the observations of Chen et al. [39] on the adaptations of desert plants. Alhawiti [59] discovered unique anticancer compounds in *H. salicornicum* due to its adaptation to the harsh desert environment.

The results indicate that desert plants are adapted to soil properties and influence the surrounding soil’s properties.

There were also significant differences between the summer and winter seasons in the carbohydrates, iron, saponin, and alkaloid content of the plants, which were higher in the winter season than in the summer. This agrees with the study of Chaves et al. [8] on seasonal variations in the chemical composition of two plants used in Brazilian traditional medicine, *Guapira graciliflora* and *Pseudobombax marginatum*, where their results showed a significant seasonal variation in the chemical composition of the extracts of both plants where some concentrations of total flavonoids and total polyphenols varied significantly between the studied seasons.

Differences in polyphenol production have also been reported in *Tulbaghia violacea*, *Hypoxis hemerocallidea*, *Merwilla plumbea*, and *Drimia robusta* plants collected in different seasons [60], and were mainly attributed to climatic differences, biological and environmental conditions, as well as genetic differences.

According to several scientific literatures, plants show a gradual increase in essential oil content during the growing season and tend to show a decrease thereafter; therefore, late summer is recommended as the best time to collect. However, other literature recommends winter as the best season for harvesting plant parts containing essential oil [61]. This is because low temperatures help in the retention of essential oils as they are highly volatile.

## 5. Conclusions

This study revealed that the growth and chemical constituents of plant communities vary during the year due to environmental and climatic changes. Availability of soil nutrients to plants depends on temperature, rain, and the microbial consortium of soil that is related to an increased growth of plants during summer. Plants of Al-Rummah valley are rich in valuable phytochemicals but their higher yield depends on the choice of season for a specific constituent. This research recommends the need to protect the region’s land more effectively, raise environmental sensitivity, cultivate plants that aerate the soil and increase its fertility, assess the environmental impact of projects or activities that may be carried out, and reduce the human influences that lead to the degradation and decline of the vegetation. Culturing medicinal and aromatic plants has a promising future if the methods and means of exploiting them are properly optimized.

## Data Availability

The original contributions presented in this study are included in the article/supplementary material. Further inquiries can be directed to the corresponding author.

## Supplementary Materials

The following supporting information can be downloaded at: https://www.mdpi.com/article/10.3390/life15030336/s1, Table S1. Analysis of variance in soil proprieties and nutrient content. Two-way ANOVA results were used to test for significant differences in means. Two variables: seasons and plant species. Values are means of triplicates. Table S2. Analysis of variance between plant species and seasons (summer and winter). Two-way ANOVA results were used to test for significant differences in means. Values are means of triplicates. Table S3. Principal Component Analysis (PCA) of the studied basic soil properties. Table S4. Principal Component Analysis (PCA) of the studied basic plant characteristics.
