# Supplementary material for: Impact of Season on Chemical Composition of Some Medicinal Plants in Saudi Arabia
Source: Life (Basel). 2025 Feb 21;15(3):336. doi: 10.3390/life15030336 (PMC11943377; doi:10.3390/life15030336)
Supplement: Supplementary file 1 [file life-15-00336-s001.zip › life-3428258-supplementary.pdf]

Table S1. Analysis of variance in soil proprieties and nutrient content. Two-way ANOVA results were used to test for significant differences in means. Two variables: seasons and plant species. Values are means of triplicates.

Season	S_P (ppm)	S_Mn (ppm)	S_Fe (ppm)	S_Mg (ppm)	S_Ca (ppm)	S_K (ppm)	S_Na (ppm)	S_N (ppm)	E.C. (dSm^{-1})	pH	S_BD ($Mg\ m^{-3}$)	SAT (%)
Summer * *C. spinose*	0.93 b	1.58 a	0.11 d	132 c	797 ab	543 a	3647 a	867 a	8.2 a	7.5 b	1.2 b	53.0 b
Summer * *H. salicornicum*	0.77 b	0.40b	0.12 d	103 c	394 c	71 b	903 b	300 bc	0.6 c	7.7 b	1.8 a	33.3 c
Summer * *Z. propinquum*	1.57 ab	1.54 a	0.11 d	132 c	786 b	502 a	4062 a	533 b	7.9 a	7.7 b	1.2 bc	54.2 ab
Winter * *C. spinose*	2.63 a	1.73 a	7.99 c	245 b	880 a	619 a	2153 ab	433 bc	3.4 bc	8.1 a	1.2 b	54.0 b
Winter * *H. salicornicum*	0.63 b	0.20 b	15.97 a	286 a	119 d	91 b	666 b	133 c	1.2 bc	8.3 a	1.8 a	33.0 c
Winter * *Z. propinquum*	2.43 a	0.44 b	13.05 b	248 b	861 ab	634 a	2284 ab	367 bc	4.8 ab	8.2 a	1.2 c	54.6 a
Pr > F (Season * Spec.)	--	*	*	*	*	--	--	--	--	--	--	--

(*) Significant differences, (-) No significant differences at $p = 0.05$.

Table S2. Analysis of variance between plant species and seasons (summer and winter). Two-way ANOVA results were used to test for significant differences in means. Values are means of triplicates.

Season	Carbohydrates	Fats	Protein (%)	Ash (%)	P_N (%)	P_P (%)	P_K (%)	P-Ca (%)	P_Mg (%)	P_Fe (ppm)	P_Cu (ppm)	P_Zn (ppm)	P_Mn (ppm)	T. Flavonoids (mg QE/g)	T. Phenolic (mg GAE/g)	T. Tannins (mgTAE/g)	T. Saponin (mgAE/g)	T. Alkaloids (mg AE/g)
Summer* *C. spinose*	16 b	3 a	10 bc	2 cd	2 bc	0.9 ab	4 a	0.8 a	0.5 ab	206 ab	36 b	105 a	68 ab	51 ab	58 b	20 ab	21 bc	60 b
Summer* *H. salicornicum*	14 b	2 a	27 a	47 a	4 a	1.0 a	3 b	0.8 a	0.5 a	184 b	259 a	25 b	53 ab	24 ab	39 b	18 b	10 d	18 b
Summer* *Z. propinquum*	13 b	2 a	29 a	28 bc	5 a	1.4 a	3 ab	0.8 a	0.3 ab	173 b	136 ab	27 b	54 ab	36 ab	144 a	21 ab	15 cd	5 b
winter* *C. spinose*	53 a	2 a	12 b	15 d	2 b	0.2 c	3 b	1.0 a	0.2 b	384 a	74 b	41 b	34 b	59 a	56 b	34 a	24 abc	348 a
winter* *H. salicornicum*	47 a	2 a	1 d	43 a	0.2 d	0.4 bc	2 b	1.0 a	0.5 ab	309 ab	10 b	11 b	42 b	34 ab	37 b	19 b	29 ab	385 a
winter* *Z. propinquum*	48 a	2 a	3 cd	32 b	0.50 cd	0.20 c	2 b	0.6 a	0.3 b	370 ab	14.22 b	17.80 b	90 a	15 b	100 ab	20 ab	34 a	168 b
Pr > F (Season*Spec.)	--	--	*	--	*	--	--	--	--	--	*	*	*	--	--	--	*	--

(*) Significant differences, (-) No significant differences at *p*= 0.05.

Table S3. Principal Component Analysis (PCA) of the studied basic soil properties.

	PC1	PC2	PC3	PC4	PC5
S_P (ppm)	0.234	**0.579**	0.183	0.000	0.003
S_Mn (ppm)	**0.723**	0.005	0.004	0.259	0.009
S_Fe (ppm)	0.279	**0.644**	0.072	0.000	0.005
S_Mg (ppm)	0.140	**0.762**	0.058	0.038	0.002
S_Ca (ppm)	**0.841**	0.081	0.060	0.009	0.009
S_K (ppm)	**0.742**	0.246	0.001	0.001	0.010
S_Na	**0.882**	0.015	0.059	0.005	0.039
S_N (ppm)	**0.743**	0.116	0.042	0.006	0.092
E.C. (dSm^{-1})	**0.814**	0.009	0.156	0.012	0.009
pH	0.271	**0.710**	0.014	0.000	0.004
S_BD ($Mg\ m^{-3}$)	**0.854**	0.139	0.000	0.006	0.000
SAT (%)	**0.854**	0.139	0.000	0.006	0.000

Table S4. Principal Component Analysis (PCA) of the studied basic plant characteristics

	PC1	PC2	PC3	PC4	PC5
Carbohydrates	**0.967**	0.001	0.024	0.002	0.007
Fats	0.128	**0.470**	0.227	0.152	0.022
Protein (%)	**0.737**	0.000	0.132	0.123	0.008
Ash	0.051	**0.569**	0.073	0.304	0.002
P_N (%)	**0.738**	0.000	0.132	0.122	0.008
P_P (%)	**0.913**	0.000	0.000	0.012	0.075
P_K (%)	0.347	**0.490**	0.136	0.006	0.020
P-Ca (%)	0.027	0.276	**0.517**	0.086	0.094
P_Mg (%)	0.334	0.036	0.000	**0.630**	0.000
P_Fe (ppm)	**0.929**	0.000	0.002	0.021	0.048
P_Cu (ppm)	**0.571**	0.023	0.258	0.002	0.147
P_Zn (ppm)	0.078	**0.531**	0.356	0.020	0.014
P_Mn (ppm)	0.001	0.251	**0.687**	0.025	0.035
T. Flavonoid (mg QE/g)	0.006	**0.972**	0.012	0.003	0.008
T. Phenolic(mg GAE/g)	0.047	0.064	0.053	**0.691**	0.144
T. Tannins(mgTAE/g)	0.200	**0.395**	0.119	0.203	0.084
T. Saponin (mgAE/g)	**0.798**	0.028	0.146	0.001	0.028
T. Alkaloid (mg AE/g)	**0.790**	0.033	0.110	0.049	0.018
